# The Role of TDP-43 in SARS-CoV-2-Related Neurodegenerative Changes

**DOI:** 10.3390/v17050724

**Published:** 2025-05-19

**Authors:** Dong-Hwi Kim, Jae-Hyeong Kim, Min-Tae Jeon, Kyu-Sung Kim, Do-Geun Kim, In-Soo Choi

**Affiliations:** 1 Department of Infectious Diseases, College of Veterinary Medicine, Konkuk University, 120 Neungdong-ro, Gwangjin-gu, Seoul 05029, Republic of Korea; opeean0@naver.com (D.-H.K.); bluetown30@naver.com (J.-H.K.); 2 Medicinal Materials Research Center, Biomedical Research Division, Korea Institute of Science and Technology (KIST), Seoul 02792, Republic of Korea; 3 Korea Brain Research Institute (KBRI), 61, Cheomdan-ro, Dong-gu, Daegu 41062, Republic of Korea; jmt1986@kbri.re.kr (M.-T.J.); kks9286@kbri.re.kr (K.-S.K.); 4 Department of Brain Sciences, Daegu Gyeongbuk Institute of Science and Technology (DGIST), Hyeonpung, Dalseong, Daegu 42988, Republic of Korea; 5 Konkuk University Zoonotic Diseases Research Center, Konkuk University, 120 Neungdong-ro, Gwangjin-gu, Seoul 05029, Republic of Korea; 6 KU Center for Animal Blood Medical Science, Konkuk University, 120 Neungdong-ro, Gwangjin-gu, Seoul 05029, Republic of Korea

**Keywords:** SARS-CoV-2, TDP-43, neurodegeneration, long COVID

## Abstract

The coronavirus disease 2019 (COVID-19) pandemic has been linked to long-term neurological effects with multifaceted complications of neurodegenerative diseases. Several studies have found that pathological changes in transactive response DNA-binding protein of 43 kDa (TDP-43) are involved in these cases. This review explores the causal interactions between severe acute respiratory syndrome coronavirus-2 (SARS-CoV-2) and TDP-43 from multiple perspectives. Some viral proteins of SARS-CoV-2 have been shown to induce pathological changes in TDP-43 through its cleavage, aggregation, and mislocalization. SARS-CoV-2 infection can cause liquid−liquid phase separation and stress granule formation, which accelerate the condensation of TDP-43, resulting in host RNA metabolism disruption. TDP-43 has been proposed to interact with SARS-CoV-2 RNA, though its role in viral replication remains to be fully elucidated. This interaction potentially facilitates viral replication, while viral-induced oxidative stress and protease activity accelerate TDP-43 pathology. Evidence from both clinical and experimental studies indicates that SARS-CoV-2 infection may contribute to long-term neurological sequelae, including amyotrophic lateral sclerosis-like and frontotemporal dementia-like features, as well as increased phosphorylated TDP-43 deposition in the central nervous system. Biomarker studies further support the link between TDP-43 dysregulation and neurological complications of long-term effects of COVID-19 (long COVID). In this review, we presented a novel integrative framework of TDP-43 pathology, bridging a gap between SARS-CoV-2 infection and mechanisms of neurodegeneration. These findings underscore the need for further research to clarify the TDP-43-related neurodegeneration underlying SARS-CoV-2 infection and to develop therapeutic strategies aimed at mitigating long-term neurological effects in patients with long COVID.

## 1. Introduction

The coronavirus disease 2019 (COVID-19) pandemic, caused by the severe acute respiratory syndrome coronavirus-2 (SARS-CoV-2), has affected global public health and resulted in worldwide social and economic challenges [1]. SARS-CoV-2 infection was initially recognized to cause severe respiratory complications, such as pneumonia and acute respiratory distress syndrome (ARDS) [2]. However, the effects of SARS-CoV-2 infection extend beyond respiratory issues, leading to complications in multiple organs, including neurological conditions affecting the central nervous system (CNS), peripheral nervous system (PNS), and skeletal muscles [3].

Transactive response DNA-binding protein of 43 kDa (TDP-43) is a nucleic acid-binding protein known to play a role in RNA processing, splicing, and transport [4]. As a member of the heterogeneous nuclear ribonucleoprotein family, TDP-43 can bind nucleic acids and proteins of neuronal cells [5]. The physiological functions of TDP-43 come from this binding capability. TDP-43 preferentially binds to UG tandem repeats located near the splice sites of pre-mRNA, regulating the splicing patterns of numerous RNA transcripts [6]. Additionally, TDP-43 interacts with non-coding RNAs, including microRNA, small nucleolar RNA, and long non-coding RNAs, playing a role in their biogenesis [7]. Based on these functions, TDP-43 is continuously involved in fundamental metabolic processes and plays a crucial role in maintaining proper cellular functioning. Under normal conditions, TDP-43 is mostly found in the nucleus; however, under pathological stress conditions, it can translocate to the cytoplasm of cells [8]. Abnormal aggregation and mislocalization of TDP-43 are associated with neurodegenerative diseases such as amyotrophic lateral sclerosis (ALS) and frontotemporal dementia (FTD) [9,10]. The pathological features of TDP-43 include ubiquitination, hyperphosphorylation, and the formation of insoluble aggregates, which can induce neuronal dysfunction and death [11]. In patients with neurodegenerative diseases, TDP-43 exhibits a misfolded structure and forms cytoplasmic inclusions, while normal cellular functions are disrupted in the absence of nuclear TDP-43 [12]. These pathogenic characteristics of TDP-43 are considered a hallmark of neurodegenerative status; therefore, the consequential links between TDP-43 abnormalities and external stressors, such as viral infections, have been increasingly studied [13].

Recent research has indicated that SARS-CoV-2 infection may induce or aggravate neurological disorders [14,15,16]. This relevance has drawn significant attention, as neurological symptoms are increasingly being recognized as a critical component of COVID-19. However, the underlying mechanisms that induce neurological complications, including those caused by TDP-43 remain unknown [13,17]. Although these studies have identified the neurological consequences of SARS-CoV-2 infection or the general roles of TDP-43 in neurodegeneration, there is a critical lack of integrative perspectives that connect these two domains closely. This review aims to fill that gap by synthesizing recent findings from virology, molecular neuroscience, and clinical studies to propose a unified framework in which SARS-CoV-2 infection may drive TDP-43 dysregulation and subsequent neurodegenerative processes. Understanding the link between SARS-CoV-2 and TDP-43 may be essential for explaining the exacerbated neurodegenerative changes observed in COVID-19 patients and may offer valuable insights into the pathological mechanisms of neurological symptoms associated with SARS-CoV-2. This review aims to examine the role of TDP-43 in the pathology of SARS-CoV-2 infection and to compile the current findings by comparing TDP-43-related neurological manifestations of COVID-19.

## 2. TDP-43 Cleavage by SARS-CoV-2 Protein

Certain SARS-CoV-2 proteins influence host cells by cleaving or modifying TDP-43. The main protease (M^pro^) of SARS-CoV-2, a highly conserved enzyme in the *Coronaviridae* family, cleaves both viral polyproteins and host cell proteins, thereby enhancing viral replication [18,19]. Since many host proteins have multiple functions, the cleavage of host proteins by M^pro^ significantly affects various biological processes. For example, cleavage of NLRP12 and TAB1 by M^pro^ contributes to the increased cytokine production and inflammatory response [20]. Specifically, NLRP12, a negative regulator of innate immunity, is cleaved by M^pro^, impairing its ability to suppress NF-κB signaling and resulting in elevated levels of pro-inflammatory cytokines such as IL-6 and TNF-α. In addition, TAB1, an essential adaptor in the MAPK/p38 pathway, is also cleaved by M^pro^, disrupting downstream signaling and further exacerbating the inflammatory milieu. These proteolytic events are implicated in the hyperinflammatory state and cytokine storm observed in severe COVID-19 patients. Cleavage of viral polyproteins is also critical for viral maturation and replication, ensuring successful viral propagation within host cells. The cleavage of virus proteins influences various viral dynamics in host cells, including immune response, cell metabolism, and intracellular transport [21]. In particular, M^pro^ cleaves TDP-43 at residue Q331 in SARS-CoV-2 infected cells, leading to aggregation and reduced solubility, which are characteristic features of TDP-43-related pathologies [22]. Mutations in TDP-43 at cleavage-prone sites often result in aggregation and cellular toxic alterations in patients with neurodegenerative diseases [23]. This suggests that M^pro^-induced TDP-43 cleavage in COVID-19 patients might affect the progression of neurodegenerative diseases. Release of the lactate dehydrogenase (LDH), a cytosolic enzyme released upon membrane damage was increased as one indicator of such toxicity. However, LDH release is only one aspect of SARS-CoV-2–induced neurotoxicity [24]. Other mechanisms which may contribute to related pathology include oxidative stress, stress granule formation, and disruption of RNA metabolism and nucleocytoplasmic transport [25,26,27,28]. The inhibition of viral protease activity can be a useful therapeutic approach for neurodegenerative diseases, as it not only disrupts viral processing and maturation but also decreases TDP-43 cleavage [29].

## 3. SARS-CoV-2 Interactions with RNA-Binding Proteins and Aggregation-Prone Proteins

The SARS-CoV-2 nucleocapsid (N) protein forms a ribonucleoprotein (RNP) complex that facilitates viral RNA translation and the formation of stress granules (SGs) and processing bodies [30,31,32]. The N protein undergoes liquid-liquid phase separation (LLPS) processes in the presence of RNA, mainly in the N-terminal intrinsically disordered regions and C-terminal oligomerization domain (CTD) [33,34,35]. SG formation through LLPS allows the viral complex of N protein and genomic RNA (gRNA) to be tightly assembled [36]. This process facilitates viral assembly and incorporates host proteins necessary for viral replication. The N protein forms biomolecular condensates with host proteins, such as TDP-43, through LLPS [37,38]. Depending on the CTD and intrinsically disordered C-terminus of TDP-43, this interaction is accelerated by specific gRNA elements [39,40,41]. N-protein quadruplex condensates incorporating the disordered domain of TDP-43 were identified in silico models [42]. The formation of TDP-43 condensates that interact with the SARS-CoV-2 N protein and gRNA can significantly affect neurodegenerative alterations and accelerate the formation of abnormal protein inclusions associated with adult-onset neurodegenerative diseases [43,44,45]. For example, sporadic ALS often involves the misfolding, aggregation, and fibrillation of TDP-43, whereas hereditary ALS involves mutations in genes such as *SOD1*, *FUS*, and *C9orf72*, all of which contribute to protein aggregation and dysfunction [46]. During SARS-CoV-2 infection, N protein incorporation inhibits the natural self-disassembly of SGs, enhancing the cellular clearance of these granules, and suppressing innate immune responses [26,47]. Nuclear magnetic resonance (NMR) studies have shown that N protein interacts with SG-related RNA-binding proteins, accelerating their phase transition to abnormal amyloid aggregation. This effect promotes the aggregation of SG-related amyloid proteins, including fused sarcoma protein (FUS), hnRNPA1, and TDP43, suggesting a correlation between SARS-CoV-2 infection and an increased risk of neurodegenerative processes due to enhanced amyloid accumulation [26]. These findings suggest that SARS-CoV-2 infection impedes SG dynamics and promotes neurodegeneration by aggregating SG-related amyloid proteins in host cells. 

Other studies have suggested that the SARS-CoV-2 S1 receptor-binding domain (RBD) binds to heparin and other heparin-binding proteins, thereby accelerating the aggregation of pathological amyloid proteins [48,49]. SARS-CoV-2 S1 RBD also interacts with aggregation-prone proteins including amyloid-beta (Aβ), alpha-synuclein (α-Syn), tau, prion proteins, and the TDP-43 RNA-recognition motif [25]. The heparin-binding site on the S1 protein may promote the aggregation of amyloid proteins on the viral surface, accelerating neurodegenerative changes. Unlike N and spike proteins, the M^pro^ protein induced increased tau aggregation over time, but did not have a similar aggregation effect for α-Syn or TDP-43 [50]. Adenosine triphosphate (ATP) was found to biphasically modulate the LLPS of the N protein, similar to its effects on human FUS and TDP-43, and to dissolve nucleic acid-induced droplets [36,51,52]. NMR data revealed that ATP binds specifically to the RNA-binding domain of the N protein, occupying a positively charged nucleic acid-binding site. These interactions may support the life cycle of SARS-CoV-2 by facilitating the RNA-handling processes [52]. The SARS-CoV-2 N protein and TDP-43 may have similar mechanisms of action in LLPS, particularly in terms of how both proteins are influenced by ATP binding to similar molecular sites. The fact that ATP modulates LLPS in a manner comparable to that of both the SARS-CoV-2 N protein and TDP-43 implies a mechanistic link between the two proteins. This connection raises the possibility that SARS-CoV-2 infection affects cellular processes that regulate pathways relevant to TDP-43 aggregation.

## 4. Molecular Interactions of SARS-CoV-2 with TDP-43

TDP-43 is an RNA-binding protein (RBP) that regulates RNA molecules and is involved in cellular RNA metabolism including splicing and translation [53]. The roles of RBPs are crucial for controlling the fate of RNA, influencing protein expression, and maintaining cellular homeostasis [54]. RBPs are also important in the viral life cycle, including in the recruitment of viral RNA to cellular membranes and in the production of subgenomic viral RNA [55,56,57]. Recent studies have suggested a relationship between TDP-43 and SARS-CoV-2, particularly in how TDP-43 might interact with the 5′ untranslated region (UTR) of the virus. As an RBP, TDP-43 binds to specific RNA sequences that are rich in UG motifs [58]. Strains of the SARS-CoV-2 from European countries exhibit single-nucleotide polymorphisms, predominantly a cytosine-to-uracil (‘C’ to ‘U’) substitution at position 241, creating a bindable site for the TDP-43 [59,60]. Structural data indicates that TDP-43 binds strongly to the UG motifs-rich region of the 5′ UTR, suggesting that its interaction with the viral RNA may enhance viral protein translation and replication. This finding highlights the fact that TDP-43 facilitates the propagation of SARS-CoV-2 within host cells, thereby contributing to its virulence [59]. Another study used the Pysster and DeepRiPe deep learning frameworks, along with cross-linking and immunoprecipitation followed by sequencing (CLIP-seq) data, to screen an in silico map of RBP-binding sites in the SARS-CoV-2 genome at a single-nucleotide resolution [61]. This analysis predicted TDP-43 binding sites in the genomic range of 89–98 within the SARS-CoV-2 wild-type reference strain. Notably, these binding sites were not found in SARS-CoV-1, MERS, or any of the other human coronaviruses analyzed, including HCoV-229E, HCoV-HKU1, HCoV-NL63, and HCoV-OC43. These findings suggest that specific TDP-43 binding in SARS-CoV-2 is a unique and newly acquired characteristic that appears to impact its virulence [61].

## 5. Long-Term Neurological Sequelae Related to COVID-19 and TDP-43 Pathology

In a mouse model using murine hepatitis virus-1 (MHV-1) coronavirus, researchers observed multiorgan pathological manifestations similar to those observed in human COVID-19 cases, with some changes persisting up to 12 months post-infection [62]. Long-term findings included severe damage to the brain, lungs, and heart, which were distinct from the changes observed in the acute phase of MHV-1 infection [63]. In the brains of MHV-1 mice, chronic changes were marked by TDP-43 hyperphosphorylation, loss of synaptic proteins, and neuronal degeneration. These changes were associated with elevated casein kinase 1 epsilon (CK1ε) levels, reduced importin-β (factors associated with TDP-43 proteinopathy), and abnormal TDP-43 and tau formations [64,65]. Notably, the inhibition of CK1ε prevented TDP-43 phosphorylation [66]. Brain injury in long-term MHV-1 infected mice was similar to the significant long-term effects of COVID-19, as evidenced by decreased levels of synaptophysin 1 that indicate a loss of synaptic integrity. This synaptic reduction may have resulted from the formation of hyperphosphorylated and aggregated TDP-43 and tau, which are implicated in neurodegeneration pathways by altering neurotransmitter and neuronal protein levels [67,68,69]. 

## 6. Association Between TDP-43-Related Neurodegenerative Disorder and SARS-CoV-2 Infection

Neurological symptoms of pre-existing neurodegenerative diseases may worsen in COVID-19 patients, and new cases of Parkinson-like features have been identified post-COVID-19 [70,71,72]. TDP-43 pathology has also been implicated in AD and PD, the two most common neurodegenerative disorders. In AD, TDP-43 inclusions are present in up to 57% of cases, particularly in the limbic system and medial temporal lobe suggesting that TDP-43 may influence the progression of AD [73,74]. Additionally, these inclusions are associated with accelerated hippocampal atrophy, cognitive decline, and co-pathology with tau and Aβ, suggesting that TDP-43 may act synergistically with classical AD pathologies to exacerbate neurodegeneration. Similarly, in PD, TDP-43 has been detected in the substantia nigra and other regions affected by Lewy body pathology [75]. Although α-Syn is the primary pathological protein in PD, TDP-43 may contribute to disease progression through mitochondrial dysfunction, impaired autophagy, and RNA dysregulation. While the direct causal role of TDP-43 in AD and PD remains to be elucidated, growing evidence suggests its involvement in broader neurodegenerative mechanisms, which could potentially be influenced or exacerbated by SARS-CoV-2 infection. Among the studies conducted, ALS and FTD are the most well-characterized diseases associated with TDP-43 pathology induced by SARS-CoV-2 infection. ALS is one of the representative neurodegenerative diseases associated with the pathological inclusions of TDP-43 and SOD1 proteins [70]. Emerging findings indicate that the SARS-CoV-2 infection may affect the pathophysiological mechanisms underlying this disorder. Both familial and sporadic ALS cases exhibit TDP-43 aggregation in motor neurons, resulting in neuronal degeneration, progressive muscle weakness, and reduced life expectancy [76,77,78,79]. SARS-CoV-2-induced oxidative stress and TDP-43 binding may induce abnormal protein formation, acting as a trigger for ALS progression from the perspective of the causative hypothesis [25,80]. Several post-COVID-19 factors accelerate TDP-43 aggregation in patients with ALS [81,82,83]. FTD is also primarily caused by the formation of abnormal proteins in the brain such as tau, TDP-43, and FUS [84,85]. TDP-43, tau, and FUS can cause neurodegenerative diseases by accumulating in the CNS as cellular aggregates that perturb cellular homeostasis. The interaction of the SARS-CoV-2 S protein with TDP-43 has been proposed to promote abnormal protein aggregation and FTD progression [25,86]. Additionally, SARS-CoV-2 infection induces redistribution of heparin-binding proteins from the axons to the soma and their subsequent hyperphosphorylation [87]. The phosphorylation of tau and TDP-43 can be modulated by the ACE2–angiotensin-(1–7)–Mas receptor axis, which normally exerts anti-inflammatory and neuroprotective effects. Activation of this pathway leads to decreased activity of pro-inflammatory kinases such as p38 MAPK and CDK5, both of which are known to phosphorylate tau and TDP-43. However, SARS-CoV-2 infection downregulates ACE2 expression by binding to and internalizing the ACE2 receptor, thereby disrupting this protective axis. As a result, the balance shifts toward the angiotensin II–AT1R axis, which promotes oxidative stress, inflammation, and kinase activation, all of which contribute to abnormal phosphorylation and aggregation of tau and TDP-43 [88,89]. These changes are implicated in neurodegenerative processes observed in patients with COVID-19 and long-term effects of COVID-19 (long COVID).

## 7. TDP-43 Pathology in Clinical COVID-19 Patients with Neurodegenerative Disorder

Human brains from patients with AD and COVID-19 show distinctive perivascular deposits of phosphorylated TDP-43 in the hippocampus and prefrontal cortex [90]. These deposits were found near the capillaries, with some colocalizing with tau and endothelial cell markers. Additionally, the abnormal accumulation of phosphorylated TDP-43 in astrocytes and hypertrophy of these cells due to SARS-CoV-2 infection are associated with more aggressive disease progression in AD [91,92]. A reduction in claudin-5 was also noted in the phosphorylated TDP-43 that had accumulated in blood vessels, indicating blood−brain barrier (BBB) disruption [93,94]. Disruption of the BBB has been observed in SARS-CoV-2 infections, as well as in vascular components related to cognitive decline and dementia in AD [95,96]. These results suggest that perivascular phosphorylated TDP-43 aggregates lead to cognitive impairment and a loss of neurovascular function in both AD and COVID-19 pathologies. Several studies have reported the detection of cytoplasmic inclusions of TDP-43 and neuropathological changes associated with encephalopathy, which is characterized by white matter hyperintensities on MRI, in elderly patients infected with COVID-19. However, the changes in TDP-43 phosphorylation/cellular localization could not be distinguished from age-related alterations [97,98]. 

## 8. Biomarker Studies of TDP-43 and Neurodegeneration in COVID-19 Patients

Biomarkers of neurological manifestations can be useful in detecting the effects of SARS-CoV-2 infection via the measurement of blood-derived proteins. Several studies have compared the changes in protein load associated with neurodegenerative diseases, including TDP-43, in neuron-enriched extracellular vesicles (nEVs) [99,100]. Markers of neuronal dysfunction were significantly elevated in nEVs among participants recovering from COVID-19 compared to historical control levels. No differences were found in the TDP-43 levels in nEVs from healthy controls and COVID-19 survivors without any long COVID symptoms. However, significantly elevated levels of TDP-43 in nEVs have been observed in patients with long COVID [99]. The detection of these proteins long after SARS-CoV-2 infection is thought to result from a positive regulatory mechanism that removes toxic proteins from the neurons. In a study examining the relationship between ACE2 protein forms and AD markers in the parietal cortex, levels of soluble ACE2 correlate positively with AD markers, such as diffuse plaques, soluble Aβ, and insoluble phosphor-tau. In contrast, membrane-bound ACE2 shows an inverse association with insoluble TDP-43 but positively correlates with soluble phosphorylated TDP-43 fragments, tau, microvascular PDGFRβ, and aminopeptidase N–suggesting that the release of ACE2 from membranes may be linked to worsening TDP-43 proteinopathy and reduced markers of BBB components [101]. In a study that tracked serum levels of neuro-injury and neurodegeneration markers in COVID-19 patients over time, the levels of Aβ42, TDP-43, and NF-L decreased during a period following hospitalization but eventually returned to baseline levels in surviving patients. Other markers, such as YKL-40 and NCAM-1, showed increased levels at specific time points. Notably, during the hospitalization period, TDP-43 levels correlated with ferritin levels, which is a marker of inflammation [102]. Collectively, TDP-43 in plasma can be utilized as a biomarker associated with neurodegenerative changes in COVID-19 patients. It can be quantified using protein quantification assays such as Western blot or ELISA, as well as commercial kits (Thermo Fisher, EPX090-15836-901). Increased levels of TDP-43 are not observed in all COVID-19 patients but are significantly increased in COVID-19 survivors with neurodegenerative symptoms. For its application as a direct neurodegenerative biomarker, quantitative assessment of the insoluble form (detergent-soluble) of TDP-43 would be particularly meaningful.

## 9. Conclusions

The COVID-19 pandemic has led to significant global health problems and economic losses. Furthermore, the multifaceted effects of SARS-CoV-2 infection affect the neurological health of patients, some for as long as 3 years after initial infection [103]. Emerging experimental and clinical studies indicate that SARS-CoV-2 infection induces or exacerbates neurodegenerative changes. RBPs such as TDP-43 are influential factors involved in neurodegenerative changes. The cleavage, aggregation, and mislocalization of TDP-43 promoted by the SARS-CoV-2 infection suggest a mechanistic link between viral infection and neurodegenerative pathologies. The dysregulation of cellular processes, including LLPS, SG dynamics, and RNA metabolism, may also be associated with TDP-43 proteinopathy (Table 1).

Understanding the intricate interactions between the SARS-CoV-2 infection and TDP-43 pathology will provide critical insights into the neurological sequelae of COVID-19. These findings emphasize the need for further research to elucidate the molecular mechanisms underlying these interactions and their long-term implications. Additional clinical and experimental data based on the correlation between TDP-43 and SARS-CoV-2 infection summarized in this review can reveal important points for preventing neuropathological damage caused by SARS-CoV-2 infection. Such efforts will not only enhance the understanding of the neurological manifestations of COVID-19, but may also prepare for therapeutic strategies for alleviating neurological sequelae in COVID-19 survivors.

## Figures and Tables

**Table 1:** Summarization of the effect of TDP-43 on SARS-CoV-2 infection and related neuropathology.

Aspects	Description	Details	References
TDP-43 cleavage	SARS-CoV-2 M^pro^ protein induces TDP-43 cleavage	**·** SARS-CoV-2 M^pro^ cleaves TDP-43 at residue Q331, leading to insolubility and aggregation. **·** Mutations in TDP-43 at cleavage-prone sites can induce neurodegenerative features.	[22,23]
LLPS formation	SARS-CoV-2 N and S proteins bind with TDP-43 promoting the formation of inclusions	**·** N-CTD and intrinsically disordered C-terminus of TDP-43 can aggregate forming abnormal inclusions associated with neurodegenerative diseases. **·** TDP-43 binds to RBD of S protein and promotes aggregation of amyloid proteins. **·** M^pro^ is not relevant to TDP-43 aggregation. **·** ATP modulates LLPS in a comparable manner for both SARS-CoV-2 N protein and TDP-43.	[31,37,42,46,47,49,67]
Molecular interaction	TDP-43 interacts with 5′ UTR of SARS-CoV-2	**·** TDP-43 binds to specific RNA sequences of SARS-CoV-2 5′ UTR that are rich in UG motifs as an RBP. **·** SARS-CoV-2 isolates from European countries exhibit single-nucleotide polymorphisms, predominantly a cytosine-to-uracil creating a bindable site for the TDP-43.	[59,60,61]
Long-term neurological sequelae	TDP-43 proteinopathy shows in COVID-19 model mouse	**·** MHV-1 infection induces long COVID-19 symptoms. **·** Elevated CK1ε levels, reduced importin-β (factors associated with TDP-43 proteinopathy), and abnormal TDP-43 and tau formations were detected in this model. **·** Brain pathology including synaptophysin 1 reduction caused by hyperphosphorylated and aggregated TDP-43.	[62]
TDP-43-related neurodegenerative disorder	SARS-CoV-2 infection affects ALS and FTD by forming inclusions of TDP-43	SARS-CoV-2 infection aggravates the symptoms in ALS patients by aggregating TDP-43 through ACE2-mediated pathway in ALS-associated brain regions Interaction of SARS-CoV-2 S protein and TDP-43 promotes abnormal protein aggregation and FTD Neurodegenerative pathway of tau and TDP-43 is typically inhibited by ACE2-related pathways, but SARS-CoV-2 infection disrupts these pathways	[97,98]
TDP-43 pathology in clinical cases	SARS-CoV-2 infection deteriorates neurodegenerative changes in AD patients	Abnormal phosphorylated TDP-43 accumulation in COVID-19 patients drives more aggressive disease progression in AD including microvasculopathy.	[90]
TDP-43 as a biomarker in COVID-19 patients	Levels of TDP-43 and related proteins can be used as biomarkers to predict the severity of neurodegenerative diseases	Levels of TDP-43 in nEVs were elevated in long COVID patients with remaining neurological symptoms. ACE2 release from membranes linked to worsen TDP-43 proteinopathy and reduced blood-brain barrier markers During the period following hospitalization TDP-43 levels decreased for a while, but eventually recovered to baseline. Ferritin levels, the marker of inflammation, are highly correlated with TDP-43 in COVID-19 patients.	[99,100,101,102]

## Data Availability

No new data were generated in the manuscript. For any inquiries, please contact the corresponding authors.

## Abbreviations

The following abbreviations are used in this manuscript:

ALS	amyotrophic lateral sclerosis
ATP	adenosine triphosphate
BBB	amyotrophic lateral sclerosis
CK1ε	casein kinase 1 epsilon
COVID-19	coronavirus disease 2019
CTD	C-terminal domain
FUS	fused sarcoma protein
FTD	frontotemporal dementia
gRNA	genomic RNA
LLPS	liquid-liquid phase separation
Long COVID	long-term effects of COVID-19
M^pro^	main protease
MHV-1	murine hepatitis virus-1
N	nucleocapsid
NMR	nuclear magnetic resonance
RBD	receptor-binding domain
RBP	RNA-binding protein
RNP	ribonucleoprotein
SG	stress granule
TDP-43	transactive response DNA-binding protein of 43 kDa
